# Eine Perspektive von Grundschulkindern auf Bedingungsfaktoren der aktiven und eigenständigen Mobilität – eine qualitative Studie

**DOI:** 10.1007/s43594-022-00080-x

**Published:** 2022-09-21

**Authors:** Selina Seemüller, Anne Kerstin Reimers, Isabel Marzi

**Affiliations:** Department für Sportwissenschaft und Sport, Friedrich-Alexander-Universität Erlangen-Nürnberg, Gebbertstr. 123b, 91058 Erlangen, Deutschland

**Keywords:** Körperliche Aktivität, Kinder, Aktiver Transport, Bedingungsfaktoren, Photovoice, Physical activity, Children, Active commuting, Determinants, Photovoice

## Abstract

Nur wenige Kinder erreichen die Bewegungsempfehlungen der Weltgesundheitsorganisation und profitieren von den gesundheitlichen Effekten der körperlichen Aktivität. Die Förderung nicht-organisierter Bewegungsaktivitäten, wie die aktive und eigenständige Mobilität, ist ein relevanter Ansatzpunkt für Interventionen zur Bewegungsförderung bei Kindern im Grundschulalter. Ziel der vorliegenden Studie war es, hinderliche und förderliche Faktoren für die eigenständige und aktive Mobilität auf dem Schulweg zu identifizieren, aus welchen geeignete Interventionsmaßnahmen abgeleitet werden können.

Im Rahmen der Studie wurden zwölf Grundschulkinder im Alter von acht bis zehn Jahren qualitativ anhand der Photovoice-Methode zu ihrem Schulweg befragt. Basierend auf dem sozial-ökologischen Modell wurden individuelle, soziale und physische Einflussfaktoren aus den Daten extrahiert. Insbesondere die elterliche Erlaubnis, eine mangelnde fahrrad- und fußgängerfreundliche Infrastruktur und rücksichtslose motorisierte Verkehrsteilnehmer*innen hindern Kinder an einem aktiven und eigenständigen Zurücklegen des Schulwegs.

Anhand der Studie wurden vielfältige Faktoren auf unterschiedlichen sozial-ökologischen Ebenen identifiziert, welche in Interventionsmaßnahmen zur Förderung eines aktiven und eigenständigen Schulwegs berücksichtigt werden sollten. Zudem sollten Verbesserungsvorschläge der Grundschüler*innen zur Ermöglichung eines aktiven und eigenständigen Schulwegs in der Stadtplanung und -entwicklung kritisch reflektiert und berücksichtigt werden, um kindgerechte Umwelten zu schaffen.

## Abstract (en)

Only a few children achieve the WHO physical activity recommendations. The promotion of non-organized physical activity, such as active and independent mobility is a relevant starting point for interventions to promote physical activity in primary school children. The aim of the present study was to identify hindering and facilitating factors for independent and active mobility on the way to school in order to guide the development of interventions.

Within the scope of the study, twelve elementary school children aged eight to ten years were qualitatively interviewed about their way to school using the photovoice method. Based on the socio-ecological model, individual, social and physical environmental factors were extracted from the data. In particular, parental permission, a lack of bicycle- and pedestrian-friendly infrastructure, and inconsiderate motorized road users hinder children from actively and independently traveling to school.

The study identified a variety of factors at different socio-ecological levels that should be considered in intervention programs to promote active and independent travel to school. In addition, suggestions made by the primary school children for improvements to enable an active and independent way to school should be critically reflected and taken into account in urban planning and development in order to provide child-friendly environments.

## Hintergrund

Bewegungsmangel ist einer der wichtigsten Indikatoren für Zivilisationskrankheiten, welche durch den technologischen und gesellschaftlichen Wandel bedingt werden (WHO 2009). Gleichwohl erreichen in Deutschland nur etwa ein Drittel der Kinder und Jugendlichen die Bewegungsempfehlungen der Weltgesundheitsorganisation (WHO 2020) im Umfang von mindestens einer Stunde moderater körperlicher Aktivität pro Tag (Burchartz et al. 2021). Zu wenig Bewegung bedingt teils schwerwiegende Folgeerkrankungen und ist mit einem erhöhten Risiko, bereits im Kindesalter an Übergewicht oder Adipositas zu erkranken, assoziiert (Janssen und LeBlanc 2010; Poitras et al. 2016). Demgegenüber können durch regelmäßige körperliche Aktivität die physische, psychosoziale und geistige Entwicklung von Kindern gefördert und gestärkt (Rütten und Pfeifer 2017; WHO 2020) sowie die Grundlagen für einen gesunden Lebensstil aufgebaut werden (Cleland et al. 2012; Telama et al. 2014). Aufgrund der hohen Prävalenz kindlichen Übergewichts einhergehend mit zahlreichen pädiatrischen Erkrankungen (Schienkiewitz et al. 2018) und aufgrund der erhöhten Morbidität und Mortalität von übergewichtigen Kindern im Erwachsenenalter (Simmonds et al. 2016) ist die Entwicklung von Maßnahmen zur Förderung von körperlicher Aktivität im Kindes- und Jugendalter ein besonderes Anliegen der öffentlichen Gesundheitsförderung (Styne et al. 2017).

**Figure 1:**
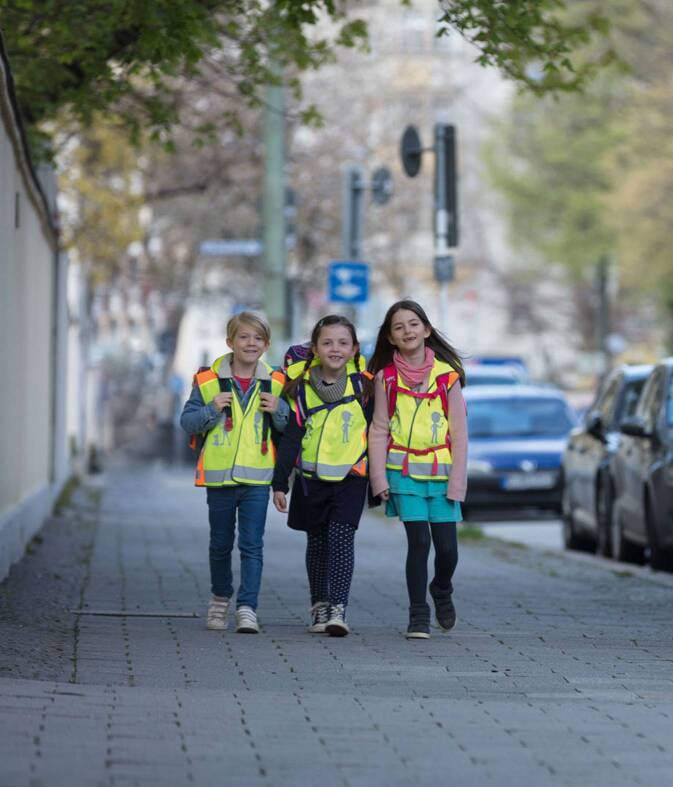

Neben organisierten Sportaktivitäten, zum Beispiel im Sportverein, können auch Alltagsaktivitäten wie das Fahrradfahren oder Zufußgehen (Abb. 1) zur Erreichung des Mindestumfangs an körperlicher Aktivität im Kindes- und Jugendalter beitragen. Diese können zudem perspektivisch, wenn sich daraus Routinen etablieren, nachhaltig und langfristig zu einem bewegungsaktiven Lebensstil auch im Erwachsenenalter beitragen (Kahlmeier et al. 2017). Wie aktuelle Daten der deutschlandweiten, repräsentativen MoMo-Studie zeigen, blieben zwar bei Kindern und Jugendlichen die Gesamtaktivität sowie die Bewegungsaktivität im organisierten Sport (Sportverein und Schulsport) über die vergangenen zehn Jahre konstant, nicht-organisierte Freizeit- und Alltagsaktivitäten wie aktiver Transport oder das Spielen im Freien nahmen jedoch um durchschnittlich 30 min pro Woche ab (Schmidt et al. 2020).

Aktuelle Studien zeigen, dass Kinder, die zur Schule laufen oder mit dem Fahrrad fahren, ein höheres Maß an körperlicher Aktivität aufweisen im Vergleich zu Kindern, die mit dem Auto oder dem Bus zur Schule gefahren werden (Chillón et al. 2010; Larouche et al. 2014). Auch belegen zahlreiche Studien einen positiven Zusammenhang zwischen dem aktiven Transport und der körperlichen Gesamtaktivität von Kindern (Kek et al. 2019; Prince et al. 2021). Zudem steht der aktive Transport in einem positiven Zusammenhang mit dem Wohlbefinden (Ramanathan et al. 2014; Stark et al. 2018) und der Eigenständigkeit von Kindern (Landwehr und Kolip 2020). Die alltägliche, eigenständige (Fort‑)Bewegung von Kindern in ihrem Wohnumfeld gilt vor allem aufgrund ihres positiven Zusammenhangs mit der Gesundheit als wichtiger Ansatzpunkt für die Bewegungsförderung (Larouche et al. 2014; Page et al. 2009; Prezza et al. 2010). Kinder, die den Schulweg aktiv und eigenständig bestreiten, erreichen beispielsweise im Durchschnitt bereits etwa 30 min (also die Hälfte) der täglichen Bewegungszeit, die in den Empfehlungen der Weltgesundheitsorganisation gefordert wird (Chillón et al. 2010). Die eigenständige und aktive Mobilität von Kindern und Jugendlichen sollte daher frühzeitig gefördert werden (Reimers et al. 2020; Reimers und Marzi 2018).

Die eigenständige Mobilität, definiert als die (Fort‑)Bewegung in der Wohnumgebung ohne die Begleitung eines Erwachsenen (Hillman et al. 1990), kann einen wesentlichen Beitrag zur Steigerung der körperlichen Aktivität von Kindern und Jugendlichen leisten (Oliver et al. 2016; Schoeppe et al. 2013). Auch wenn diese Definition auch die eigenständige Nutzung des öffentlichen Personennahverkehrs einschließt, wird die eigenständige Mobilität in der Mehrheit der empirischen Studien mit einem aktiven Zurücklegen einer Wegstrecke zu Fuß oder mit dem Fahrrad assoziiert. Aus gesundheitswissenschaftlicher Sicht wird die eigenständige Mobilität entsprechend als gesundheitsrelevantes Verhalten in der Kindheit definiert, welches die eigenständige Fortbewegung im Wohnumfeld von Kindern ohne elterliche Aufsicht umfasst (Reimers und Marzi 2018).

Auch wenn in Studien ähnliche gesundheitliche Vorteile für die aktive Mobilität und die eigenständige Mobilität berichtet werden (Garcia-Cervantes et al. 2016; Larouche et al. 2020), ist es von entscheidender Bedeutung, beide Verhaltensweisen zu adressieren. Im Gegensatz zur aktiven Mobilität mit Begleitung eines Erwachsenen zeigen Kinder, die auf dem Schulweg ohne elterliche Begleitung unterwegs sind, ein höheres Maß täglicher körperlicher Aktivität (Mackett et al. 2007; Richard et al. 2020). Neben einer Steigerung der körperlichen Aktivität eröffnet ein hohes Ausmaß an eigenständiger Mobilität Kindern zudem den Zugang zu verschiedenen Erfahrungs‑, Spiel- und Sozialräumen (Reimers und Marzi 2018). Außerdem zählen neben der Förderung der Gesundheit geringere Kosten für öffentliche oder private motorisierte Verkehrsmittel und die Schonung der Umwelt durch Vermeidung von CO_2_-Emissionen, welche durch weniger motorisierten Straßenverkehr erzielt wird, zu den Vorteilen der eigenständigen, aktiven Mobilität von Kindern (Brand et al. 2021).

Aufgrund des Rückgangs der eigenständigen Mobilität von Kindern in den vergangenen Jahrzehnten ist die Entwicklung und Implementierung von Interventionen, welche die eigenständige und aktive Mobilität von Kindern fördern, notwendig (Reimers und Marzi 2018). In Deutschland ist die Anzahl der Grundschulkinder, die den Schulweg eigenständig bestreiten, zwischen 1990 und 2010 von 93 % auf 76 % gesunken (Shaw et al. 2013). Ähnliche Trends zeigen sich weltweit auch in anderen Nationen (Reimers und Marzi 2018; Shaw et al. 2015).

Zur Entwicklung von Maßnahmen zur Förderung der eigenständigen Mobilität bedarf es an Erkenntnissen darüber, welche Bedarfe und Bedürfnisse damit einhergehen und welche Faktoren das Verhalten fördern oder hemmen können. Das sozial-ökologische Modell von Sallis et al. (2006) liefert eine gute Strukturierungsgrundlage hinsichtlich individueller, sozialer und umweltbezogener Determinanten von körperlichen Aktivitäten wie der eigenständigen Mobilität. Der sozial-ökologische Ansatz beinhaltet die Annahme, dass Gesundheitsverhalten durch unterschiedliche Faktoren, welche verschiedenen Ebenen zuzuordnen sind, bedingt wird und Wechselwirkungen zwischen den Faktoren verschiedener Ebenen bestehen. Zu den Einflussfaktoren zählen Faktoren der individuellen Ebene (zum Beispiel soziodemographische Faktoren und persönliche Einstellungen), des sozialen Umfeldes (zum Beispiel Unterstützungsverhalten der Eltern, Wahrnehmung von Sicherheit und Verkehr), der baulichen Umwelt (zum Beispiel Infrastruktur) sowie der politische Ebene (zum Beispiel Schulpolitik) (Giles-Corti et al. 2005; Sallis et al. 2006).

Auch wenn aus zahlreichen Studien zu Determinanten der eigenständigen Mobilität bereits erste Ansatzpunkte für Interventionen hervorgehen (Marzi et al. 2018), fehlen aktuell Studien zur Kinderperspektive hinsichtlich der hinderlichen und förderlichen Faktoren der eigenständigen Mobilität (Marzi und Reimers 2018). Der Fokus lag in bisherigen Forschungsarbeiten meist auf der Sicht der Eltern und die Kinderperspektive wurde bisher kaum berücksichtigt (Marzi und Reimers 2018), obwohl beide Perspektiven erheblich voneinander abweichen können und für die Entwicklung von Interventionen beiderseits relevant sind. Beispielsweise fanden Crawford et al. (2017) in einer qualitativen Befragung von Kindern und deren Eltern heraus, dass Kinder von einem breiteren Spektrum an Sicherheitsbedenken als ihre Eltern berichteten, zum Beispiel durch die Schikane durch andere Kinder oder aufgrund von Dunkelheit. Auch in Bezug auf die Verkehrsmittel für den Schulweg unterscheiden sich die Präferenzen und Einstellungen zwischen Eltern und Kindern (Kohler und Kreipl 2002).

Im deutschsprachigen Raum liegen aktuell nur wenige Studien zur eigenständigen Mobilität vor (Reimers und Marzi 2018). Auch werden Kinder selten partizipativ in Interventionen oder in die Gestaltung von Maßnahmen der Gesundheitsförderung eingebunden und werden selten nach ihrer Sicht gefragt (Kolip und Finne 2018; Larsson et al. 2018). In aktuellen Debatten werden deshalb verstärkt partizipative Forschungs- und Planungsmethoden gefordert (King et al. 2016; Kolip und Finne 2018). Ähnliches postuliert auch die UN-Kinderrechtskonvention, welche das Mitbestimmungsrecht von Kindern sowie die Teilhabe am gesellschaftlichen Leben und damit einhergehend an Veränderungen fordert (Reitz 2015; United Nations 1989).

Daher ist das Ziel der vorliegenden Studie, die Bedingungsfaktoren für einen eigenständigen und aktiven Schulweg von Grundschulkindern aus ihren individuellen Sichtweisen zu erfassen, um geeignete kindgerechte Interventionsmaßnahmen ableiten zu können. Um die subjektiven Determinanten valide zu erfassen, wird die Photovoice-Methode zur qualitativen und partizipativen Datenerhebung genutzt. Die Determinanten der eigenständigen Mobilität aus Sicht der Grundschulkinder werden anhand der Ebenen des sozial-ökologischen Modells eingeordnet (Giles-Corti et al. 2005; Sallis et al. 2006). Dieser Beitrag soll Akteur*innen des Gesundheits- und Schulsystems sowie der Stadt- und Infrastrukturplanung Anhaltspunkte zur Implementierung von Interventionen zur Förderung der aktiven und eigenständigen Mobilität von Grundschulkindern geben.

## Methodik

Um die Bedingungsfaktoren eines aktiven und eigenständigen Schulwegs aus der Sicht von Kindern zu erforschen, wurde die qualitative, partizipative Forschungsmethode Photovoice angewendet (Layh et al. 2020; Wang und Burris 1997), in Anlehnung an Landwehr und Kolip (2020). Grundgegenstand der Forschungsmethode stellen die individuell angefertigten Bilder zu einem gesellschaftsrelevanten Thema dar, welche im Nachgang in Gruppeninterviews diskutiert werden. Damit weist diese Methode einen entscheidenden Unterschied zu anderen qualitativen Forschungsmethoden auf, welche auf professionell angefertigten Fotografien beruhen. Ein weiterer Unterschied zu anderen partizipativen Forschungsmethoden bildet die Intention von Photovoice-Studien, welche Informationen generieren, die für Interventionen genutzt werden können (Wihofszky et al. 2020). Die in den 1990er-Jahren von Wang und Burris (1997) konzipierte Methode wird vor allem in der qualitativen Sozialforschung aufgrund des hohen Maßes der Beteiligung der Proband*innen an der Datenerhebung und -auswertung vielfach genutzt (Hergenrather et al. 2009). Anhand der Visualisierung von Geschehnissen durch die Aufnahme von Bildern können Zusammenhänge und Aspekte ausgedrückt und thematisiert werden, welche schwer versprachlicht werden können (von Unger 2013; Weber 2008). Die zugehörigen Fragen in den Gruppeninterviews bieten den Teilnehmenden Denkanstöße (Wang und Burris 1997).

Für die Studie wurden Kinder im Alter zwischen acht und zehn Jahren aus einer fränkischen Mittelstadt (circa 77.000 Einwohner*innen) rekrutiert. Die Rekrutierung erfolgte durch Kontaktaufnahme mit einer Grundschule. Die weitere Vorgehensweise (unter anderem Studiendurchführung, teilnehmende Klasse, Kontaktaufnahme mit den Eltern) wurde mit dem Direktorat der Schule sowie einer Klassenlehrkraft besprochen. Die Studie wurde durch ein Informationsschreiben den Eltern und Kindern vorgestellt und das Einverständnis zur Teilnahme, sowohl der Eltern als auch der Kinder, eingeholt. Auch über datenschutzrechtliche Aspekte wurde informiert. Zu Beginn der Studie wurden die Kinder gebeten, einen Fragebogen zur Erfassung ihres Alters, ihres Geschlechts, der Dauer des Schulwegs, des hauptsächlich genutzten Verkehrsmittels und der Schulwegbegleitung auszufüllen. Fünf Tage vor den geplanten Interviews erhielten die Kinder folgende Aufgabenstellung: „*Mache drei Fotos Deines Schulwegs. Achte hierbei darauf, Stellen zu fotografieren, die Dir helfen oder Dich daran hindern, Deinen Schulweg zu Fuß, mit dem Fahrrad oder dem Roller alleine (ohne die Begleitung von Erwachsenen) zurückzulegen.“ *Der zeitliche Rahmen von fünf Tagen zur Anfertigung der Fotos wurde ausgewählt, um auf den Erinnerungen der Kinder aufbauen zu können. Vor allem bei Kindern im befragten Alter ist dieser zeitliche Rahmen ein wichtiges, zu beachtendes Merkmal (Reinders 2016). Die Fotos wurden im Anschluss per Mail durch die Eltern der Studienleitung zugestellt, sodass sie zum Interviewtermin vorlagen.

Aufgrund der Covid-19-Pandemie fanden die Gruppeninterviews online über die Online-Video Software Zoom (Zoom Video Communications, Inc., San Jose, USA, verfügbar unter https://zoom.us/) statt. Die Gruppenzusammenstellung von vier Dreiergruppen erfolgte aufgrund einer erhöhten Redebeteiligung und Motivation bei Kleingruppen (Zwick und Schröter 2012) und der Empfehlung der Einteilung in altershomogene Kleingruppen (Vogl 2015). Die Gruppeninterviews wurden von der Studienleitung moderiert, welche die Kinder begrüßte und nochmals das Einverständnis für die auditive Aufzeichnung des Interviews von den Kindern einholte.

Für die Gruppeninterviews wurden vorab Regeln aufgestellt. Diese besagten beispielsweise, dass sich die Kinder gegenseitig ausreden lassen sollten, damit alle Kinder sich frei äußern, mitentscheiden und gleichberechtigt an der Diskussion teilhaben konnten. Im Interview wurden alle Kinder dazu aufgefordert, ihr Lieblingsfoto auszuwählen, zu präsentieren und allen Teilnehmenden der Gruppe etwas darüber zu erzählen (Nykiforuk et al. 2011). Durch das Teilen des Bildschirms hatten alle Kinder immer das aktuell besprochene Foto vor Augen. Die Interviews dauerten zwischen 31 und 37 min und wurden auf der Basis des SHOWeD-Verfahrens (Wang 1999) gestaltet und hinsichtlich der Forschungsfrage auf folgende Leitfragen angepasst: Was siehst Du auf dem Foto? Was wurde hier fotografiert? Was passiert hier wirklich? Wie beeinflusst Dich die Stelle in Deinem Schulweg? Was sollte sich hier ändern?

Im direkten Anschluss an die Interviews wurden diese vollständig im Wortlaut transkribiert. Um die Interviews zu pseudonymisieren, wurden die Namen der Kinder durch Buchstaben ersetzt. Die Auswertung der Interviews erfolgte nach der qualitativen Inhaltsanalyse nach Mayring (2020). Durch das kategoriengeleitete Vorgehen wurde die Auswertung des Interviews auf die wesentlichen Bedeutungsaspekte der verbalen Aussagen beschränkt und paraverbale Informationen wurden vernachlässigt, da diese für die Informationsentnahme hinsichtlich der vorliegenden Fragestellung nebensächlich sind (Reinders 2016). Die Codierung der Transkripte erfolgte zunächst deduktiv, in Anlehnung an die Ebenen des sozial-ökologischen Modells (Giles-Corti et al. 2005; Sallis et al. 2006). Die anschließende induktive Kategorienbildung erfolgt durch die Zusammenfassung analytischer Kategorien über alle Transkripte hinweg. Die Bedingungsfaktoren der eigenständigen und aktiven Mobilität von Grundschulkindern wurden hinsichtlich förderlicher und hinderlicher Faktoren und der von den Kindern geäußerten Verbesserungsvorschläge eines aktiven, eigenständigen Schulwegs gegliedert.

## Ergebnisse

### Merkmale der Stichprobe

Die teilnehmenden Grundschüler*innen (*N* = 12) waren je zur Hälfte Jungen und Mädchen (Tab. 1). Die Altersspanne der Kinder reichte von acht bis zehn Jahren. Die Schüler*innen wohnen aufgrund des sehr großen Einzugsgebiets der Schule in verschiedenen Stadtteilen und benötigen für ihren Schulweg in einfacher Richtung zwischen fünf und dreißig Minuten. Über die Hälfte der befragten Kinder kommt mit dem Bus oder Auto zur Schule. Fünf Kinder nutzen aktive Verkehrsmittel, wobei drei Kinder zur Schule laufen und drei Fahrrad fahren. Die meisten Kinder kommen ohne Begleitung oder mit anderen Kindern zur Schule, lediglich zwei Kinder werden auf dem Schulweg von einer erwachsenen Person begleitet.

**Table 1:** 

Alter (MW ± SD)		8,5 ± 0,65 Jahre
Geschlecht	Weiblich	6
	Männlich	6
Hauptverkehrsmittel	Bus	6
	Auto	1
	Fahrrad	2
	Zu Fuß	3
Begleitung	Keine	5
	Anderes Kind	5
	Erwachsene Person	2
Schulwegdauer (MW ± SD)		14,9 ± 10,1 min

In Anlehnung an den sozial-ökologischen Ansatz (Giles-Corti et al. 2005; Sallis et al. 2006) werden im Folgenden die Bedingungsfaktoren, welche die Kinder in ihrer Verkehrsmittelwahl hinsichtlich einer aktiven, eigenständigen Bestreitung des Schulwegs beeinflussen, dargestellt. Aufgrund der Wechselbeziehungen zwischen den Ebenen und den thematisierten Faktoren in den Interviews erfolgt die Kategorisierung der Ergebnisse anhand von drei Ebenen: individuelle Faktoren, Faktoren der sozialen Umwelt und Faktoren der physischen Umwelt.

### Individuelle Faktoren

In Bezug auf die individuumsbezogenen Einflussfaktoren thematisierten die interviewten Kindern insbesondere motivationale Aspekte sowie die Einstellung gegenüber der aktiven und eigenständigen Mobilität auf dem Schulweg als förderliche Faktoren. Im Gegensatz dazu stellte sich in den Interviews heraus, dass fehlendes Wissen über Straßenverkehrsregeln und das eigene Kompetenzempfinden die Kinder davon abhält, den Schulweg aktiv, vor allem mit dem Fahrrad, zu bestreiten.

#### Einstellung und Motivation zu Bewegung

Die Kinder äußern eine grundlegende Freude an Bewegung und Eigenständigkeit durch das selbstständige und aktive Zurücklegen von Schulwegen. Auch formulieren die Kinder positive Effekte von aktiven Schulwegen:

In der Schule sitzt man eh schon so viel. Also ich würde schon gerne mit dem Roller oder Fahrrad oder so fahren. Ich hätte da schon Lust drauf. (Kind G, m, Bus)

Die meisten Kinder bewerten auch die generelle, eigenständige Mobilität, welche sie in ihrer Freizeit erleben, als positiv. Einige Kinder haben diese Erfahrung auch bereits auf den Schulweg übertragen und schätzen die Unabhängigkeit von ihren Eltern oder anderen erwachsenen Personen.

Und ich fahre auch immer zu meinen Freunden mit dem Fahrrad. Dann kann ich ja auch zur Schule fahren. (Kind H, w, Fahrrad)

Das eigenständige Erreichen von Spielplätzen, Freund*innen oder andere Freizeitaktivitäten nach der Schule fördert die Entscheidung für das Fahrradfahren zur Schule. Die Möglichkeit, eigene Entscheidungen zu treffen, beispielsweise nach der Schule eine*n Freund*in zu besuchen, oder zu einem Spielplatz zu fahren, unabhängig von Busfahrzeiten oder elterlichen Vorgaben, nehmen die Kinder an der eigenständigen, aktiven Mobilität positiv wahr:

Ich komme immer an dem Spielplatz vorbei, wenn ich mit dem Fahrrad zur Schule fahre. Und ich finde es gut, dann kann ich selbst entscheiden, wie lange ich dableibe, weil ich mich nicht an Buszeiten halten muss. (Kind L, w, Fahrrad)

Diese Spontanität und Eigenständigkeit motiviert die Kinder, zur Schule zu laufen oder mit dem Fahrrad zu fahren, anstatt den Bus zu nehmen. Die gewonnene Flexibilität durch die Nutzung aktiver Verkehrsmittel macht einen aktiven Schulweg für die Kinder attraktiver:

Und ich will nicht Bus fahren oder so. Weil nach der Schule fahre ich dann oft noch mit zu einer Freundin, wenn die Mama arbeitet. Und das könnte ich ja sonst nicht. (Kind H, w, Fahrrad)

#### Kompetenz und Wissen

Auch wenn die interviewten Kinder grundsätzlich gerne aktiv und eigenständig zur Schule kommen würden und die positiven Aspekte der Bewegung schätzen, hindert sie eine fehlende Verkehrserziehung daran, öfter und eigenständig mit dem Fahrrad zur Schule zu kommen. Da die Verkehrserziehung an bayerischen Grundschulen erst in der vierten Klasse erfolgt, fehlt den Kindern notwendiges Wissen über das Verhalten im Straßenverkehr (Bayerisches Staatsministerium für Unterricht und Kultus 2022). Die erworbenen Kompetenzen und das Wissen über Verkehrsregeln würden ihnen helfen, sich im Straßenverkehr sicherer zu fühlen:

Meine Schwester hat mal gesagt, da lernt man auch so viel über den Verkehr. Also wie man sich verhalten soll, wenn man auf der Straße ist. Das finde ich eigentlich jetzt schon wichtig. (Kind F, m, zu Fuß)

**Figure 2:**
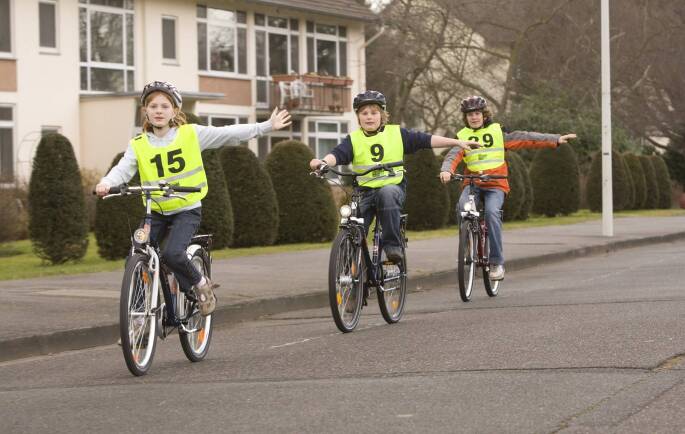

Zudem hindert die fehlende Verkehrsausbildung auch die Eltern daran, ihren Kindern den Schulweg mit dem Fahrrad, dem Roller oder zu Fuß zu erlauben. Dabei wurde zum einen die rechtliche Erlaubnis, mit dem Fahrrad zur Schule zu kommen, in Frage gestellt, als auch darüber diskutiert, ob das Fahrradfahren zur Schule ohne einen Fahrradführerschein (Abb. 2) erlaubt ist. Entsprechend dürfen die Kinder aufgrund des fehlenden Fahrradführerscheins noch nicht selbstständig mit dem Fahrrad zur Schule fahren:

Aber alleine darf ich gar nicht fahren wegen dem Fahrradführerschein, den habe ich ja noch nicht. (Kind A, w, Bus)

Aus Sicht der Kinder wäre es deshalb gut, die Verkehrserziehung und den Fahrradführerschein schon zu einem früheren Zeitpunkt in der Schule zu thematisieren beziehungsweise zu erwerben.

### Faktoren der sozialen Umwelt

Das Bedürfnis, den Weg zur Schule eigenständig und aktiv zu bestreiten, ist bei vielen der befragten Kinder groß. Jedoch wird diese Möglichkeit nicht nur von individuellen Faktoren, sondern auch durch das soziale Umfeld, insbesondere die Eltern, beeinflusst. In den Interviews wurden die Eltern, welche bei Grundschulkindern als zentrale Bezugspersonen gelten, als wesentlicher Einflussfaktor der sozialen Umwelt identifiziert. Neben der generellen elterlichen Erlaubnis, den Schulweg aktiv und eigenständig zu bestreiten, thematisierten die interviewten Kinder die Begleitung durch Erwachsene oder andere Kinder als entscheidend für das Fahrradfahren auf dem Schulweg.

Die elterliche Erlaubnis wurde insbesondere in Bezug auf das Fahrradfahren ohne erwachsene Begleitung thematisiert. Während eine Begleitung beim Busfahren oder Zufußgehen nur in seltenen Fällen als notwendig erachtet wurde, fehlt bei vielen Kindern die elterliche Erlaubnis, eigenständig den Schulweg mit dem Fahrrad zu bestreiten:

Der Mama ist das [Fahrradfahren] meistens zu gefährlich und mir auch. Deswegen fahr ich dann meistens Bus. (Kind G, m, Bus)

Obwohl die Eltern aus zeitlichen Gründen zum Beispiel durch die Berufstätigkeit ihr Kind nicht immer begleiten können, ist die elterliche Begleitung auf dem Schulweg aufgrund von Sicherheitsbedenken der Eltern teilweise notwendig:

Manchmal laufe ich zur Schule, aber manchmal haben meine Eltern auch Angst. […] Manchmal kommt die Mama dann mit, dann laufen wir zusammen. (Kind F, m, zu Fuß)

Aus Sicht der Kinder können aktive Schulwege durch die Begleitung von anderen Erwachsene (Eltern anderer Kinder) oder von anderen Kindern ermöglicht werden:

Ich darf nur Fahrrad fahren, wenn ein Erwachsener dabei ist, also manchmal fährt die Mama von [Person B] mit, dann geht es […]. Aber alleine darf ich gar nicht fahren […]. (Person A, w, Bus)

Wenn ich mal mitfahren darf, wenn deine Mama fährt, dann würde ich das machen. Und dann können wir ja noch andere Kinder mitnehmen. (Kind C, m, Bus)

### Faktoren der physischen Umwelt

In Hinblick auf die physische Umwelt wurden die Infrastruktur, Sicherheitsaspekte wie bauliche Strukturen und andere Verkehrsteilnehmer*innen sowie die Entfernung als bedeutende Faktoren für die eigenständige und aktive Mobilität aus der Kinderperspektive identifiziert.

#### Infrastruktur

Infrastrukturelle Bedingungen wie zum Beispiel Fahrradwege sind aus Sicht der Kinder aktuell nicht ausreichend gegeben. Die Kinder äußern klare Vorstellungen über die Gestaltung der Infrastruktur für aktive Schulwege. Vor allem die Verbesserung der Beschaffenheit der Wege, welche häufig in desolatem Zustand sind, sowie die Trennung von nicht-motorisiertem und motorisiertem Verkehr sind Bedingungen für das Fahrradfahren:

Ich würde aber gerne mit dem Fahrrad zur Schule fahren, aber ich müsste ganz lange auf einer großen Straße fahren. Daneben ist zwar ein Radweg, aber der ist ganz kaputt. Da sind überall Löcher. Und auf der Straße sind zu viele Autos, da habe ich Angst, dass die den Abstand nicht einhalten. (Kind D, w, Bus)

Dabei ist für die Kinder eine Trennung von Rad- und Fußweg nicht so bedeutsam, sondern vielmehr ein Ausbau und die Instandsetzung der Fahrradwege.

#### Sicherheit

Weitere bauliche Strukturen auf dem Schulweg wie uneinsichtige Kreuzungen, zum Beispiel durch eine hohe Mauer, welche Kindern die Sicht auf den Verkehr versperrt, führen bei den Kindern zu einem Gefühl der Unsicherheit im Straßenverkehr. Auch große Kreuzungen mit einem hohen Verkehrsaufkommen führen zu Ängsten und Unsicherheiten bei Eltern und Kindern:

Und da rasen immer die Autos so. Und die passen auch nie auf, also manche bloß. Und die bleiben dann auch nicht stehen. Und fahren einfach weiter. Und da muss ich jeden Tag rüber und muss aufpassen. (Kind H, w, Fahrrad)

Auch eine Unterführung wurde von den Kindern als hinderlich für den aktiven Schulweg erachtet, da diese zu eng für Radfahrer*innen sei und es keine ausreichende Beleuchtung gäbe. Die Kinder fühlen sich dadurch unsicher und möchten nicht alleine durch die Unterführung laufen oder mit dem Fahrrad hindurchfahren. Unter diesen Umständen präferieren die Kinder motorisierte Verkehrsmittel:

Ich fühle mich mit dem Bus sicherer. Weil da bei der Unterführung, da ist es so eng, sagt auch die Mama. (Kind C, m, Bus)

In Hinblick auf die Unterführung äußern die Kinder klare Verbesserungsvorschläge, um das eigenständige Durchqueren zu ermöglichen und ihre Sicherheitsbedenken zu reduzieren:

Also es wäre ja schon gut, wenn da ein Licht wenigstens wäre. Dann wäre es nicht so dunkel. Und wenn der Fußgängerweg vielleicht bisschen breiter wäre. (Kind G, m, Bus)

Zudem entwickelten die Kinder die Idee einer Brücke oder eines Weges für Radfahrer*innen zur Überquerung der Schienen sowie eines Ampelsystems für die Züge:

Kann man da nicht so einen Weg drübermachen? (Kind I, m, zu Fuß)

Ja, aber da fahren ja Züge drüber. (Kind G, m, Bus)

Ja, aber es gibt ja auch Ampeln für Züge. (Kind I, m, zu Fuß)

Zentral für das Sicherheitsempfinden von Grundschulkindern im Straßenverkehr und die Einschränkung der aktiven und eigenständigen Mobilität sind aus Sicht der Kinder andere Verkehrsteilnehmer*innen. Ein Unterschreiten von Mindestabständen und das Missachten von Zebrastreifen und Tempolimits sowie das hohe Verkehrsaufkommen auf dem Schulweg hindern die Kinder an einem eigenständigen und aktiven Schulweg. Die Kinder berichten von Schwierigkeiten, Zebrastreifen gefahrlos zu überqueren, wenn diese von Autofahrer*innen missachtet werden:

Aber ich muss halt immer da über den Zebrastreifen und die Autos halten sich da nicht dran. Weil eigentlich müssten die ja anhalten. Machen die aber voll oft nicht. (Kind E, w, Auto)

Zudem äußern die Kinder die Angst, dass Autofahrer*innen auch eine rote Ampel überfahren würden. Um besonders rücksichtslose Autofahrer*innen dazu zu zwingen, sich an die Straßenregeln zu halten, entwickelten die Kinder verschiedene Ideen:

Da müsste noch was sein, was die Autos davon abhalten würde, zu fahren. (Kind F, m, zu Fuß)

Höchstens eine Schranke vielleicht, aber das gibt es ja nirgendwo. (Kind D, w, Bus)

Aber wenn da so ein Drücker an der Ampel wäre, dass die Ampel gleich grün wird, das wäre besser. Oder ein Schulweghelfer. (Kind. F, m, zu Fuß)

Um andere Verkehrsteilnehmer*innen auf fahrradfahrende Grundschulkinder aufmerksam zu machen, betonen einzelne Kinder in den Interviews, dass Straßen fahrradfreundlicher gestaltet werden sollten:

Ja, also das Problem ist auch, dass man ja dann links abbiegen muss zur Schule, und da überholen die Autos dann einfach, obwohl man Handzeichen gibt. (Kind C, m, Bus)

Ja, man könnte das ja am Boden markieren, dass die Autos das wissen. (Kind B, w, Bus)

Ja, die haben ja jetzt auch ein neues Schild hingemacht. Dass man beim Fahrradfahren nicht überholt werden darf. (Kind C, m, Bus)

Das Sicherheitsempfinden im Straßenverkehr wird zudem durch die notwendige Konzentration und die damit verbundene Anstrengung bei hohem Verkehrsaufkommen eingeschränkt:

Mit dem Fahrrad darf ich ja eh noch nicht zur Schule fahren und das will ich auch nicht, weil das ist eh immer so anstrengend, weil mit den Autos da muss man da immer so aufpassen. (Kind E, w, Auto)

#### Entfernung

Als weiteren Aspekt der physischen Umwelt nannten die Kinder die Länge der zurückzulegenden Wegstrecke. Die Distanz zur Schule, verbunden mit einem erhöhten zeitlichen Aufwand und einem frühen morgendlichen Aufstehen, wurde als generelle Barriere eines aktiven, eigenständigen Schulwegs identifiziert. Weite Distanzen vom Elternhaus zur Schule führen dazu, dass Kinder eher motorisierte Verkehrsmittel nutzen:

Also ich wohne halt voll weit weg von der Schule. Mit dem Bus fahr ich immer schon so 20 min. Und mit dem Fahrrad würde das ja noch länger dauern und dann müsste ich noch früher aufstehen. (Kind J, m, Bus)

## Diskussion

Ziel der vorliegenden Studie war es, Bedingungsfaktoren für einen eigenständigen und aktiven Schulweg von Grundschulkindern aus der Kinderperspektive zu erfassen. Die Kinder diskutierten verschiedene Möglichkeiten und Grenzen aktiver Schulwege, welche auf Basis des sozial-ökologischen Modells nach Giles-Corti et al. (2005) zusammengefasst wurden. Dabei kamen Faktoren auf der individuellen Ebene und auf der Ebene der sozialen und der physischen Umwelt zur Sprache. Insbesondere die fehlende Erlaubnis von Eltern, eine mangelhafte Fahrradinfrastruktur sowie rücksichtslose Verkehrsteilnehmer*innen hindern Kinder daran, ohne die Begleitung von Erwachsenen mit dem Fahrrad oder zu Fuß zur Schule zu gelangen. Demgegenüber berichten die Kinder von Freude, Motivation und einem positiven Erleben von Selbstständigkeit in Bezug auf aktive und eigenständige Schulwege. Aus den Interviews der Kinder wurde zudem die Interaktion der verschiedenen Faktoren aus unterschiedlichen Einflussebenen deutlich (Sallis et al. 2006). So wurden beispielsweise zahlreiche bauliche Maßnahmen genannt, die sowohl die Sicherheitsbedenken der Kinder als auch der Eltern minimieren und die eigenständige und aktive Mobilität fördern könnten.

### Individuelle Faktoren

**Figure 3:**
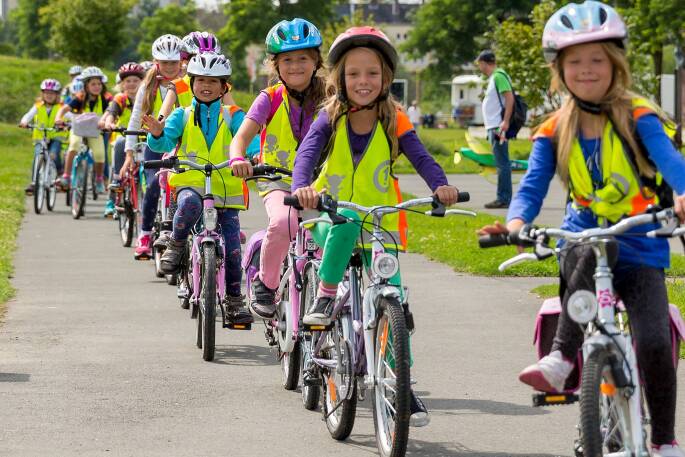

In der Studie stellte sich ein starkes Bedürfnis der Kinder nach einem eigenständigen, aktiven Schulweg heraus. Das eigenständige Erreichen der Schule ist bei vielen Kindern auch mit dem Erreichen anderer Freizeitaktivitäten, Freund*innen oder Institutionen assoziiert und hat für die Kinder eine hohe Relevanz. Wie aktuelle Forschungsarbeiten bestätigen, erwerben Kinder durch die eigenständige Mobilität im Wohnumfeld ein höheres Maß an Selbstständigkeit (Bittmann 2008) und lernen, sich in der Wohnumgebung zu orientieren (Rissotto und Tonucci 2002). Auch wenn lediglich zwei der interviewten Kinder mit dem Fahrrad zur Schule fahren, stand das Fahrradfahren im Fokus der Interviews. Neben gesundheitlichen Aspekten wie einer erhöhten körperlichen Aktivität beim Radfahren gegenüber dem Zufußgehen (Larouche et al. 2014) gewinnt das Fahrradfahren gesellschaftlich auch aus umwelt- und verkehrspolitischer Sicht an Bedeutung (Abb. 3) (Brand et al. 2021; Bundesministerium für Verkehr und digitale Infrastruktur 2021). Trotz der Motivation Fahrrad zu fahren, hindern eine fehlende Fahrkompetenz sowie fehlendes Wissen über Verkehrsregeln Kinder daran, alleine mit dem Fahrrad zur Schule zu fahren. Wie eine Studie mit Eltern von Kindern im Alter von neun bis 15 Jahren zeigt, ist auch die elterliche Einschätzung der Kompetenzen ihres Kindes, den Schulweg sicher bestreiten zu können, ausschlaggebend für die eigenständige Mobilität (Bennetts et al. 2018).

### Faktoren der sozialen Umwelt

Die Interviews mit den Kindern zeigen deutlich, dass Kinder den Schulweg häufig aufgrund elterlicher Vorgaben nicht eigenständig und aktiv zurücklegen. Aus Sicht der Kinder hindern insbesondere Sicherheitsbedenken die Eltern daran, ihren Kindern die eigenständige Mobilität zu erlauben. Befragungen von Eltern zeigen, dass die Angst um das Kind meist im Straßenverkehr begründet ist (Bennetts et al. 2018; Rauh et al. 1995). Dies führt dazu, dass Eltern ihren Kinder nur eingeschränkt erlauben, sich frei in ihrer Umgebung zu bewegen (Limbourg und Reiter 2003). Eine Begleitung der Kinder durch die eigenen Eltern, andere Erwachsene oder andere Kinder stellt eine Möglichkeit dar, den Schulweg aktiv (und möglicherweise auch ohne Begleitung durch Erwachsene) zu bestreiten, wie die vorliegende Studie zeigt. Die von den Kindern in den Interviews entwickelte Idee eines „cycling school bus“ wird international bereits in einer Vielzahl von Interventionsstudien in ähnlicher Form (als „walking school bus“) als effektive Maßnahme zur Steigerung des aktiven und eigenständigen Schulwegs beschrieben (Brettschneider und Malek 2005; Farella et al. 2020; Smith et al. 2015; Yang et al. 2014). An verschiedenen Haltestellen können sich Kinder zu vorgegebenen „Abfahrtszeiten“ dem aktiven (laufenden) Bus anschließen und auf einer festen Route (gegebenenfalls in Begleitung eines Erwachsenen) gemeinsam zur Schule laufen (Brettschneider und Malek 2005). Prinzipiell wäre dieses Konzept gleichermaßen als Interventionsmaßnahme zur Förderung des Radfahrens zur Schule denkbar.

### Faktoren der physischen Umwelt

Abgesehen von der Begleitung fordern die Kinder mehr Sicherheit im Straßenverkehr, die Trennung von Radwegen und Straßen und die Einhaltung der Verkehrsregeln durch Autofahrer*innen. Die Nichteinhaltung geltender Regeln wird von den Kindern als gefährdend empfunden und hindert sie an der aktiven, eigenständigen Teilhabe am Straßenverkehr. Die Kinder berichteten darüber hinaus, dass für ihre Eltern vor allem die mangelnde Sicherheit durch eine fahrrad- und fußgängerunfreundliche Infrastruktur sowie durch andere Verkehrsteilnehmer*innen bedeutend sei. Die Forschungsergebnisse dieser Studie sowie Befunde anderer Studien und Interventionen in Bereichen der Nahmobilität von Grundschulkindern bestätigen diese elterlichen Sicherheitsbedenken (Bennetts et al. 2018; Landwehr und Kolip 2021; Marzi et al. 2018; Zougheibe et al. 2021). Eine quantitative Befragung in der Großstadt Essen ergab, dass 70 % der Kinder Gefahrenpunkte auf ihrem Schulweg benennen können, welche sich vor allem auf das Verhalten anderer Verkehrsteilnehmer*innen beziehen (Limbourg und Reiter 2003). In den vorliegenden Gruppeninterviews schlugen die Kinder die Installation von Geschwindigkeitsüberwachungen (Blitzer) und Warnschildern als Maßnahmen vor. Darüber hinaus könnten ihnen zufolge eine stärkere Polizeipräsenz beziehungsweise Schüler*innenlotsen, Blitzerampeln und mehr Kontrollen an Zebrastreifen für mehr Sicherheit auf dem Schulweg sorgen.

Neben Maßnahmen in Bezug auf andere Verkehrsteilnehmer*innen empfanden die Kinder ihre Umgebung als eher fahrradunfreundlich, vor allem aufgrund der unzureichend ausgebauten Infrastruktur, welche sich im schlechten Zustand von Fahrradwegen und nicht klar abgetrennten Fahrradwegen manifestiert. Durch Ausbesserung dieser fußgänger- und fahrradunfreundlichen Infrastruktur könnten auch Weglängen- und Zeitvorteile für aktive Verkehrsteilnehmer*innen ermöglicht werden, beispielsweise durch die damit verbundene Verkürzung der Wege und weniger Wartezeit an Straßenampeln (Hackl et al. 2019; Landwehr und Kolip 2021). Die Sicherheit auf dem Schulweg, beispielsweise durch eine ausreichende Beleuchtung, ist ein wichtiger Faktor für die eigenständige Mobilität von Kindern (Carver et al. 2014). Aktuelle Studien belegen, dass infrastrukturelle Maßnahmen zur Förderung aktiver Schulwege beitragen können (Chen et al. 2018).

### Wechselwirkungen zwischen den sozial-ökologischen Ebenen

Neben der isolierten Betrachtung der unterschiedlichen Ebenen des sozial-ökologischen Modells geht aus den Interviews der Kinder hervor, dass einzelne Bedingungsfaktoren eines aktiven, eigenständigen Schulwegs in zahlreichen Wechselbeziehungen mit anderen Ebenen des Modells stehen. Am Beispiel des Faktors Sicherheitsbedenken der Schüler*innen lassen sich diese Interaktionen erkennen und die Komplexität der Bedingungsfaktoren der aktiven und eigenständigen Mobilität im Grundschulalter verdeutlichen. Im Fokus stehen die intrapersonal empfundene Angst und Unsicherheit im Straßenverkehr, welche die Kinder daran hindert, ihren Schulweg aktiv und eigenständig zu bestreiten. Auf diesen zentralen Bedingungsfaktor haben zahlreiche weitere politische, umweltpolitische, soziale und infrastrukturelle Bedingungen einen Einfluss. Die genannte Verkehrserziehung, welche an bayerischen Grundschulen in der vierten Klasse im Lehrplan verankert ist, hilft den Kindern, ein sicherheitsbewusstes Handeln im Verkehr zu erlernen und kann Sicherheitsbedenken gegenüber der Aktivität und Eigenständigkeit im Straßenverkehr mindern. Ebenso werden die Bedenken der Kinder durch elterliche Verbote und Regeln beeinflusst. Die bestehenden Sicherheitsbedenken der Eltern können jedoch ebenfalls auf einen fehlenden Fahrradführerschein beziehungsweise fehlende Verkehrserziehung zurückgeführt werden. Zuletzt beeinflussen auch das Verhalten anderer am Verkehr beteiligter Personen, mangelnde Kontrollen dieses Verhaltens sowie mangelnde bewegungsfreundliche Infrastruktur das Sicherheitsgefühl der Grundschüler*innen und schränken die aktive und eigenständige Mobilität ein.

### Implikationen

Die durchgeführte explorative Studie gibt erste Hinweise auf die Bedürfnisse der Kinder für die eigenständige und aktive Mobilität. Diese identifizierten Bedingungsfaktoren sollten in Planungen für eine bewegungs- und fahrradfreundlichere Stadt aufgenommen und berücksichtigt werden. Neben einer angemessenen Gestaltung von Interventionen zur Bewegungsförderung gilt es zu klären, warum Kinder ihrem eigentlichen Wunsch nach eigenständiger, aktiver Bewegung auf ihrem Schulweg nicht nachkommen (können). Wie die aktuelle Studie zeigt, wird neben eigenen Sicherheitsbedenken der Kinder und fehlender Infrastruktur den Eltern eine zentrale Rolle im Entscheidungsprozess zugeschrieben. Sowohl die Kinder- als auch die Elternperspektive sollte bei der Planung von Maßnahmen einbezogen werden. Interventionen, die die Infrastruktur betreffen, können dazu beitragen, dass sich Kinder auf ihrem Schulweg sicherer fühlen und öfter aktiv und eigenständig zur Schule kommen.

Es besteht Handlungsbedarf auf mehreren Ebenen des sozial-ökologischen Modells (Giles-Corti et al. 2005), da diese sich wechselseitig beeinflussen. Beispielsweise könnten infrastrukturelle Maßnahmen, wie ein Ausbau der Fahrradinfrastruktur verbunden mit Verkehrsschulungen für Kinder, die Sicherheitsbedenken von Eltern und Kindern senken und die eigenständige und aktive Mobilität dadurch fördern (Bennetts et al. 2018). Die Studienergebnisse geben erste Erkenntnisse und Ansätze für die Förderung der aktiven und eigenständigen Mobilität von Kindern und machen die notwendige Differenzierung der Eltern- und Kinderperspektive deutlich. Weitere Forschungsvorhaben sollten größere Stichproben einschließen, um die Repräsentativität der Ergebnisse kritisch zu prüfen.

### Stärken und Schwächen der Studie

Die vorliegende Studie zeichnet sich durch den partizipativen Ansatz der Forschungsmethode Photovoice für die Erhebung der kindlichen Bedürfnisse aus. Die Photovoice-Methode ist in ihrer Durchführung zeit- und ressourcenaufwändig, generiert jedoch wichtige Informationen aus der Sichtweise von Kindern. Diese Informationen können für eine zielgruppengerechte Maßnahmenplanung genutzt werden. Durch die Fotografien wurde umfangreiches Gesprächsmaterial bereitgestellt, welches in den Interviews genutzt wurde und zur Gestaltung der Interviews beigetragen hat. Innerhalb der Interviews wurde den Kindern durch diese Methode ein hoher Grad an Partizipation ermöglicht. Besondere Bedeutung erhält die Studie aufgrund der Erfassung der kindlichen Bedürfnisse sowie ihrer Einschätzungen und Vorschläge. Um die aktuelle Forschungslücke der kindlichen Perspektive auf Bedingungsfaktoren der eigenständigen und aktiven Mobilität zu adressieren, wurden die Eltern nicht in das Forschungsvorhaben eingeschlossen. Allerdings ist die elterliche Mitwirkung und Lenkung bei der Erstellung und Auswahl der Fotomotive aufgrund des Alters der teilnehmenden Kinder nicht auszuschließen. Da das Familienumfeld und insbesondere die Eltern eine zentrale Rolle im Entscheidungsprozess zur Verkehrsmittelwahl von Kindern einnehmen (Panter et al. 2008), könnten zukünftige Forschungsvorhaben davon profitieren, beide Perspektiven zu berücksichtigen, um den elterlichen und kindlichen Einfluss auf das Mobilitätsverhalten gegenüberzustellen. Als weitere Schwäche der Studie ist das Online-Format der Gruppeninterviews zu nennen. Aufgrund der Durchführung im Online-Format sind möglicherweise Hemmnisse aufgetreten, die die Kinder davon abhielten, ihre Meinung zu äußern. Die Durchführung weiterer Photovoice-Studien in anderen Städten und anderen Klassenstufen könnte dazu beitragen, die vorliegenden Ergebnisse zu bestätigen und weitere Implikationen für Forschung und Praxis abzuleiten.

## Fazit

Die eigenständige und aktive Mobilität fördert die kindliche Entwicklung und eröffnet Kindern eine Vielzahl an Erfahrung‑, Sozial- und Spielräumen (Marzi und Reimers 2018). Ziel der vorliegenden Studie war es, Bedingungsfaktoren für eine eigenständige und aktive Bestreitung des Schulwegs aus Sicht von Grundschulkindern basierend auf dem sozial-ökologischen Modell (Giles-Corti et al. 2005) zu erfassen. Aus der individuellen Perspektive von Grundschulkindern wurden anhand der Photovoice-Methode förderliche und hinderliche Faktoren diskutiert und Verbesserungsvorschläge aus Sicht der Kinder erarbeitet. Die Studie identifizierte eine Vielzahl individueller, sozialer und physischer Einflussfaktoren, welche in Interventionsmaßnahmen zur Förderung des aktiven und eigenständigen Schulwegs adressiert werden sollten. Aus der vorliegende Studie geht hervor, dass Kommunen und Schulen durch gezielte Maßnahmen dazu beitragen können, dass sich Kinder auf ihrem aktiven und eigenständigen Schulweg sicherer fühlen, beispielsweise durch den vermehrten Einsatz von Verkehrshelfer*innen, welche Kindern beim Überqueren von Ampeln oder Zebrastreifen helfen. Eine dauerhaft installierte Geschwindigkeitsüberwachung vor Grundschulen könnte helfen, Geschwindigkeitsüberschreitungen zu verhindern. Solche Maßnahmen verbessern die Einhaltung von Straßenverkehrsregeln, können zu einem rücksichtsvolleren Umgang unter Verkehrsteilnehmer*innen führen und können das Sicherheitsgefühl der Schüler*innen stärken (Gehlert und Kröling 2018). Die Verbesserungsvorschläge der Kinder zur Ermöglichung eines aktiven und eigenständigen Schulwegs sollten in der Stadtplanung und -entwicklung kritisch reflektiert und berücksichtigt werden, um kindgerechte Umwelten für eine aktive Mobilität zu schaffen.
